# Association between perceived exertion and executive functions with serve accuracy among male university tennis players: A pilot study

**DOI:** 10.3389/fpsyg.2023.1007928

**Published:** 2023-01-25

**Authors:** Yuta Kuroda, Toru Ishihara, Masao Mizuno

**Affiliations:** ^1^Department of Sport Education, Hokusho University, Ebetsu, Hokkaido, Japan; ^2^Graduate School of Education, Hokkaido University, Sapporo, Japan; ^3^Graduate School of Human Development and Environment, Kobe University, Kobe, Japan; ^4^Faculty of Health and Medical Care, Hachinohe Gakuin University, Hachinohe, Japan

**Keywords:** cognitive function, physical exertion, racquet sports, sports performance, second serve

## Abstract

Serve in tennis is a very important strokes and is positively correlated with the rankings of the Association of Tennis Professionals ranking. This study investigated the associations between time-course changes in the ratings for perceived exertion, executive function, and second serve accuracy during 30-min tennis exercise sessions. Eleven Japanese male tennis players participated in the study, and their executive function and second serve performance were evaluated using the paper version of the Stroop Color and Word Test, followed by a serve performance test. The participants took part in a 30-min tennis exercise program and performed the Stroop Color and Word Test, heart rate (HR) check, and second serve accuracy test before and after the tennis exercise. Pearson correlation was used to determine the relationships between the ratings for perceived exertion, interference scores on Stroop Color and Word Test performance, and second serve performance. Post exercise, the rating of perceived exertion tended to correlate with serve accuracy (*r* = −0.57, *p* = 0.07) and interference score (*r* = 0.65, *p* = 0.03). The pre-to-post changes in second serve accuracy were negatively associated with the changes in interference score (*r* = −0.54, *p* = 0.08) and interference score in the posttest (*r* = −0.73, *p* = 0.01). The results suggest that time-course changes in executive function when playing tennis are positively associated with the accuracy of the second serve. These findings expand the previous knowledge regarding the positive association between time-course changes in executive functions and percentage of points won when playing tennis by including more specific skills (i.e., second serve accuracy).

## 1. Introduction

There is a positive association between sports performance and cognitive function at rest (Vestberg et al., 2012; Alexandru et al., 2014 Sakamoto et al., 2018; Ishihara et al., 2019). This line of literature focused on executive functions, which include goal-directed cognitive processes that coordinate and regulate thought and action. For example, previous findings suggested that greater executive function at rest predicts future success in tennis (Ishihara et al., 2019) and soccer (Vestberg et al., 2012; Sakamoto et al., 2018). However, studies on the association between time-course changes in executive function and performance while playing sports is scarce. Since executive function is altered by acute exercise (Ishihara et al., 2021), evidence of the association between executive function and sports performance during matches will benefit players and their coaches.

Ishihara et al. (2018) investigated the relationship between match statistics variables and executive function in singles tennis matches. The findings showed that executive function evaluated during matches was positively correlated with the percentage of points won. Additionally, executive function during tennis singles matches was inversely correlated with perceived exertion. However, this previous study focused on relationship between tennis match analysis and cognitive function, and the possible association between time-course changes in executive function and more specific skills remains unclear.

A serve refers to one of the strokes the players can hit at their own time, and this behavior is called self-paced. It is classified into categories similar to those of pitching in baseball, bowling, golfing, and running. However, the action affected by the opponent’s movement is called externally paced. Soccer, basketball, and volleyball are classified in a similar category (Singer, 2000). Functions that support higher cognitive functions such as decision-making, problem solving, and inhibitory control are called executive functions, and exercise has a particularly strong association with executive functions (Diamond, 2013). Jacobson and Matthaeus (2014) examined the characteristics of executive function (e.g., decision-making, problem solving, and inhibitory control) of an externally paced athlete, a self-paced athlete, and a non-exercising college student. They reported that inhibitory control is more important in a self-paced athlete compared to externally paced athletes and non-exercising college students. Furthermore, among self-paced athletes, those with high athletic performance have high inhibitory control. Inhibitory control is the ability to control attention, behavior, thought, and emotion to suppress unnecessary information inside or outside as well as selectively focus on important information (Diamond, 2013). In tennis competitions, athletes must ensure that the second serve stays within the service box. Therefore, the second serve is a shot focusing on accuracy. When players hit the second serve, it is necessary to suppress the unnecessary attention on audiences and opponents and pay attention to the service box. Therefore, inhibitory function is significant in the accuracy required for the second serve.

In modern tennis, competitive players must maintain their serve quality throughout the game to win a match. Serves account for 45, 60, and 56% of all strokes played during service games in the French Open, Wimbledon, and US Open, respectively (Johnson et al., 2006). Furthermore, the numbers of aces, valid first serves, and second serve points won were associated with winning the match in the Association of Tennis Professionals (ATP) dataset includes 18,288 performances from 9,144 matches involving 845 players held between 1991 and 2008 (Ma et al., 2013). Moreover, the number of double faults in the Grand Slam has been decreasing year by year (Cross and Pollard, 2009). Winning the point on the second serve (*r* = −0.64) is more related to higher ATP rankings than winning the point on the first serve (*r* = −0.28; Reid et al., 2010). Considering the importance of second serve performance in winning tennis matches, examining the contribution of executive function to second serve performance during matches warrants clarification. Hence, this study investigated the associations among time-course changes in the rating of perceived exertion, executive function, and second serve accuracy during 30-min tennis exercise sessions.

## 2. Methods

### 2.1. Subjects

Eleven men participated in this study, including seven from the All Japan Student Tennis Championship (mean [range]: age: 23.1 years [19–30]; height: 175.5 cm [166.8–183.0]; weight: 65.1 kg [53.8–74.0]). The All Japan Student Tennis Championship is a tournament open only to the top few winners of the regional qualifying rounds in each prefecture. Therefore, the participants are athletes with high skill levels. All of them were right-handed. Participants provided written informed consent in accordance with the requirements of the Hokusho University Research Ethics Committee (HOKUSHO-UNIV:2020-006). This study was approved by Hokusho University Research Ethics Committee. They were instructed to consume their daily intake on the day before and the day of the exercise, avoid alcohol intake the day before the exercise, and avoid caffeine and smoking on the day of the exercise.

### 2.2. Experimental protocol

Participants were asked to remain quiet upon arriving at the experimental site. They wore a heart rate (HR) monitor. After resting for 5 min, resting heart rate was measured and resting executive function was then measured at pre training exercise. After completing the executive function measurement, participants performed a normal warm-up, subsequently the first second serve accuracy test was performed. Participants undertook 30 min of training exercise (10 min ground strokes vs. ground strokes rally, with 20 min volley vs. strokes rally). Our preliminary experiments have demonstrated that this 30-min exercise, which is a habitual practice in tennis, can be performed with relatively high intensity. This exercise was used in the present study to examine the relationship between service accuracy and executive function under conditions that were less stressful than the actual match (removing the tactical component). They were allowed unlimited water intake every 10 min during the exercise. The rating of perceived exertion (RPE) was measured immediately post the exercise using the Borg scale. After 30 min of training exercise, the executive function and the second serve accuracy test at post training exercise were measured following the same procedure as that at pre training exercise.

### 2.3. Measurement

#### 2.3.1. Executive function assessment

Participants’ basic cognitive processing and inhibitory control were assessed using a modified version of the Stroop Color and Word Test (SCWT; Stroop, 1935), that comprises two trials (congruent and incongruent). The stimuli used in the congruent trials were four colored patches (red, yellow, green, and blue). The incongruent trials used color names as stimuli, where the color of the text did not match the named color (e.g., the word “BLUE” was printed in yellow). The stimuli for each task appeared on a gray background. For the congruent condition, 48 patches (vertical 8 × horizontal 6) were printed in red, green, blue, or yellow at random. All participants read these patches. For the incongruent condition, the words “RED,” “GREEN,” “BLUE,” or “YELLOW” were printed in an incongruent color on 48 patches. The word stimuli were presented in Japanese. Participants were required to quickly respond to the two conditions. The main variables were the differences in the two conditions (*Interference score* in reaction time [RT] and correct reactions), using the mean RT and correct reactions of each trial, which were calculated as follows (Scarpina and Tagini, 2017):


$$\text{Interference score}=\left(\text{incongruent–congruent}\right)/\text{congruent}\times 100.$$

The interference score is evaluated as the amount of interference increases when the numerical value increases. This amount of interference evaluates the inhibitory control at SCWT, and if the value increases before and after exercise, it can be considered that the inhibitory function has decreased. In this study, we examined time-course change in interference score pre and post exercise.

#### 2.3.2. Rating of perceived exertion

The rating of perceived exertion (RPE) was measured immediately post the exercise using the Borg (1982) scale. After the exercise, participants were asked, “How difficult was the exercise?” The Borg scale assessed their perceived physical exertion on a scale of 6 to 20.

#### 2.3.3. Heart rate

HR was measured using a Heart Trainer (Konami, Japan). Exercise intensity was calculated from the preliminary HR (%HRR) using the HR pre and post the exercise:


MaximumHR=220–Age

$$\;\;\text{HRR}=\left(\text{HR}–\text{resting}\;\text{HR}\right)/\left(\text{maximum}\;\text{HR}–\text{resting}\;\text{HR}\right)\times 100.$$

#### 2.3.4. Serve accuracy performance test

**Figure 1:**
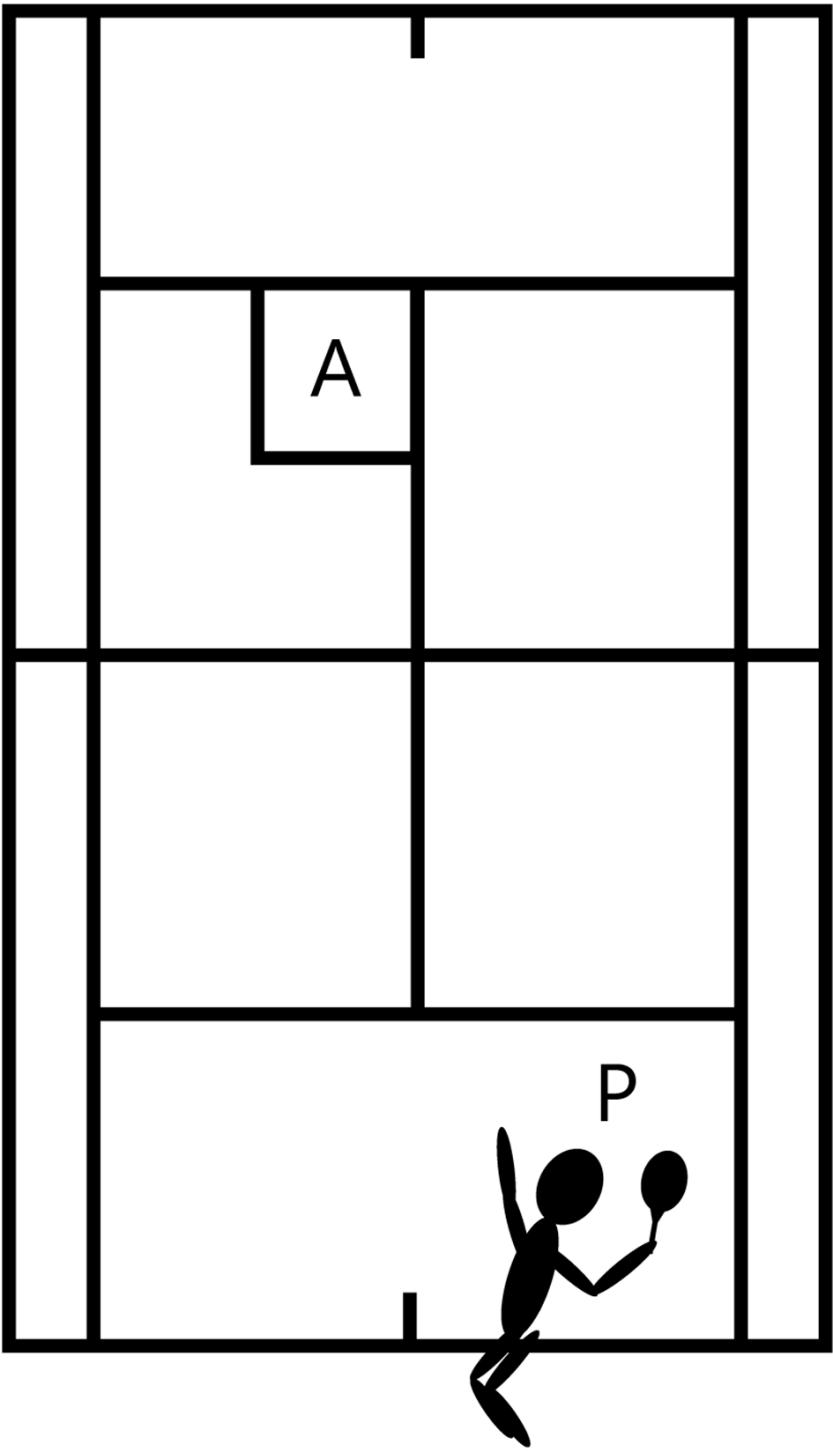
Serve accuracy. The subjects served 20 times from point P to the left side of the serve box, aiming at target A. Serve accuracy was determined as the number of serves that successfully hit target A out of 20 serves. The players hit their second serves at match pace [A: target zone [1.07 m × 1.07 m]; P: the player].

Serve accuracy is determined by counting the number of times the ball landed within the designated target zone —that is, serves that landed in a hitting area located in the back-right region of the service box (1.07 m × 1.07 m, the height of singles sticks; see Figure 1; Kuroda et al., 2016). This test used only the deuce side and required the players to hit 20 trial second serves. The serve accuracy performance test was performed before and after the exercise, and the success of each serve was decided by two referees who were tournament-level tennis players. In this study, we examined time-course change in serve accuracy pre and post exercise.


$$\begin{array}{l} \text{Change in serve accuracy}\\ =\text{serve accuracy performance test}\;\text{at}\;\text{post exercise}\\ –\text{serve accuracy performance test}\;\text{at}\;\text{pre}\;\text{exercise} \end{array}$$

#### 2.3.5. Statistical analysis

The results of the normality test were *p* < 0.18 and *p* < 0.34 for the Kolmogorov–Smirnov and Jarque-Bera tests, respectively. The time-dependent change in each measurement item was analyzed using a paired *t*-test. Effect sizes are shown in Cohen’s d. Serve accuracy and cognitive functions, as well as their relationship with other measurement items, were evaluated using Pearson’s correlation coefficient. The statistical significance was defined as *p* < 0.05. Given the sample size of 11 participants and a power = 0.80, the present study theoretically had sufficient sensitivity to detect an effect size exceeding *r* = 0.68 for the variance explained by each variable in the regression analysis (G*Power 3.1; Faul et al., 2007, 2009).

## 3. Result

The intensity of the exercise, result of SCWT, and second serve accuracy are presented in Table 1.

**Table 1:** The intensity of the exercise, result of SCWT, and second serve accuracy.

	Pre		Post	
	Mean ± SD	Range	Mean ± SD	Range
Stroop color word task				
Congruent condition reaction time (sec)	22.8 ± 3.4	17.8–29.0	21.2 ± 3.3	17.5–29.8
Congruent condition correct reactions (%)	96.2 ± 3.3	89.6–100	95.1 ± 4.3	85.4–100.0
Incongruent condition reaction time (sec)	32.3 ± 5.1	23.3–39.7	31.6 ± 6.4	22.7–45.9
Incongruent condition correct reactions (%)	91.5 ± 4.7	85.4–97.9	85.6 ± 7.4	70.8–93.8
Interference score reaction time (%)	42.2 ± 15.0	18.4–64.7	49.0 ± 16.0	23.8–71.1
Interference score correct reactions (%)	-4.9 ± 4.2	−12.8–0.0	−10.0 ± 6.6	−23.4–2.3
Serve accuracy performance test				
2nd serve accuracy (times)	6.0 ± 1.3	5.0–8.0	5.4 ± 2.1	3.0–10.0

### 3.1. Rating of perceive exertion and HR

The participants’ average rating of perceive exertion was 15.4 immediately post the exercise (standard deviation [SD] = 1.8) as measured on the Borg scale. The mean HR were 68.7 ± 9.9 bpm and 147.1 ± 16.9 bpm at pre and post exercise, respectively. The mean percentage of HRR calculated was 51.7 ± 11.5%.

### 3.2. Executive function

The mean SCWT RTs in the congruent trials were 22.8 s (SD = 3.4) pre exercise and 21.2 s (SD = 3.3) post exercise, which were significantly different [*t*(10) = 2.52, *p* = 0.03, Cohen’s d = 0.50]. The mean SCWT RTs in the incongruent trials were 32.3 s (SD = 5.1) pre exercise and 31.6 s (SD = 6.4) post exercise, which were also not significantly different [*t*(10) = 0.57, *p* = 0.57, Cohen’s d = 0.14]. Additionally, interference score at pre and post were 42.2% (SD = 15.0) and 49.0% (SD = 16.0), respectively; which did not change significantly [*t*(10) = −1.29, *p* = 0.23, Cohen’s d = −0.44; Table 1].

The rate of correct reactions in the congruent trials pre and post exercise were 96.2 (SD = 3.3) % and 91.5 (SD = 4.7) %, respectively; which did not change significantly [*t*(10) = 1.15, *p* = 0.27, Cohen’s d = 0.29]. The rate of correct reactions in the incongruent trials pre and post exercise were 95.1 (SD = 4.3) % and 85.6 (SD = 7.4) %, respectively, constituting a significant decrease post exercise [*t*(10) = 3.76, *p* = 0.004, Cohen’s d = 0.95]. The interference scores at pre and post exercise were-4.9% (SD = 4.2) and-10.0% (SD = 6.6), respectively, constituting a significant change post-exercise [*t*(10) = 2.57, *p* = 0.03, Cohen’s d = −0.44; Table 1].

### 3.3. Serve accuracy

The number of times the ball landed within the designated target pre and post exercise were 6.0 times (SD = 1.3) and 5.4 times (SD = 2.1), respectively. Thus, serve accuracy did not differ significantly pre and post exercise, and seven of the 11 players had decreased accuracy in their serves post the exercise [*t*(10) = 0.78, *p* = 0.45, Cohen’s d = 0.37].

### 3.4. Relationship between RPE, interference score, and serve accuracy

**Figure 2:**
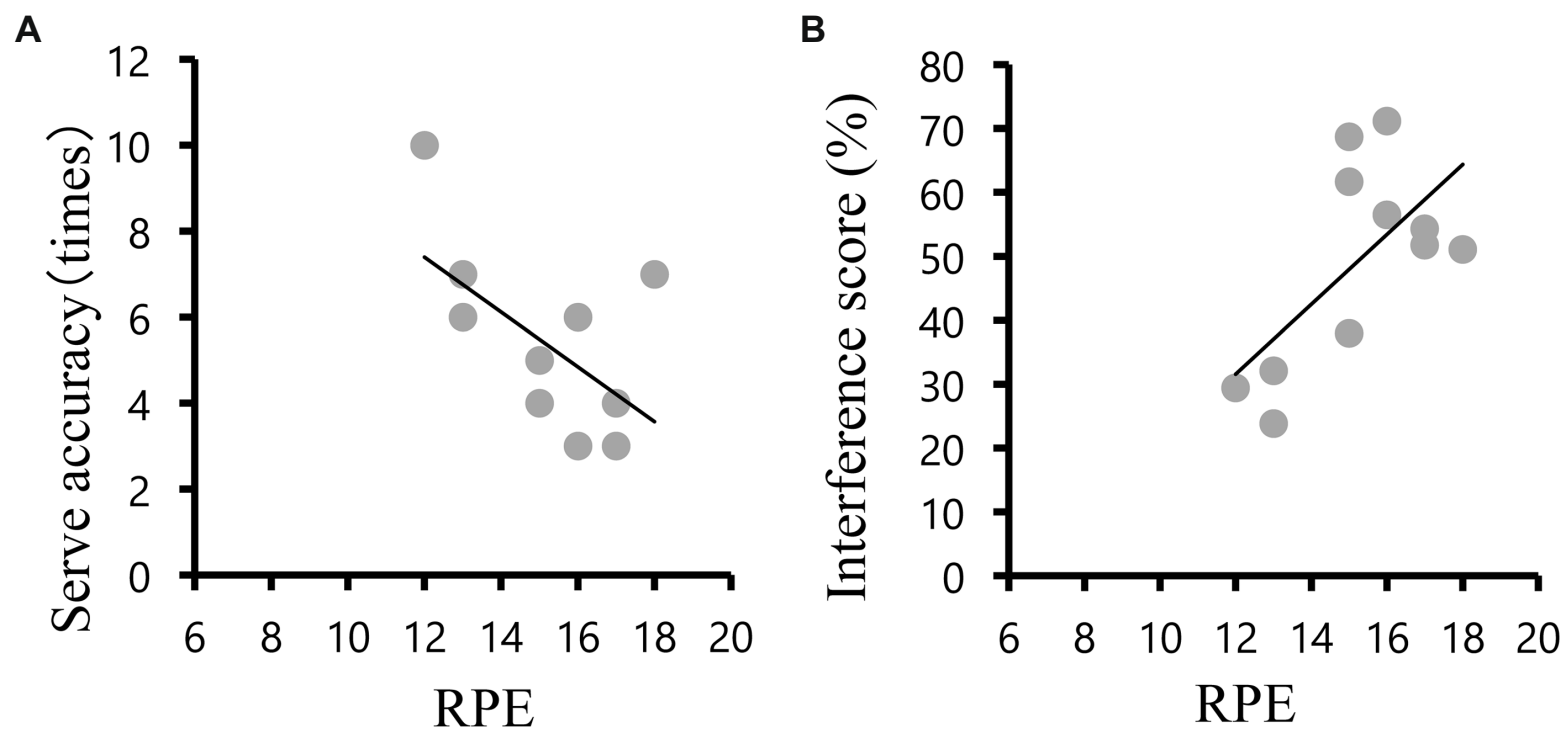
**(A)** An inverse correlation was detected between post-exercise serve accuracy and post-exercise rating of perceived exertion (*r* = −0.57, *p* = 0.07). **(B)** A correlation was detected between post-exercise reaction time in the Stroop Color and Word Test and post-exercise rating of perceived exertion (*r* = 0.65, *p* = 0.03).

**Figure 3:**
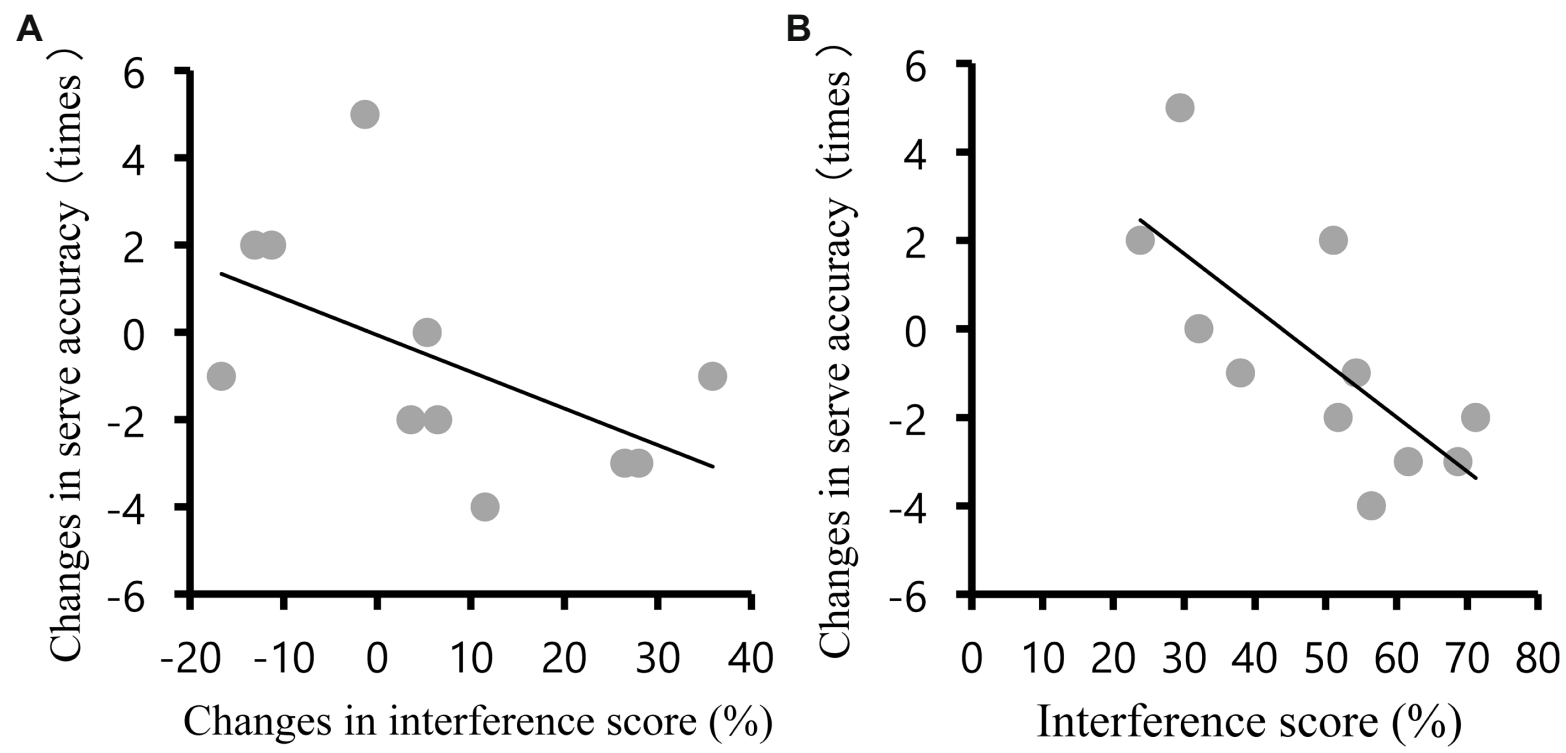
**(A)** The pre-to-post changes in second serve accuracy were negatively associated with the changes in interference score of RT in the posttest (*r* = −0.54, *p* = 0.08). **(B)** The pre-to-post changes in second serve accuracy were negatively associated with the interference score of RT in the posttest (*r* = −0.73, *p* = 0.01).

The relationship between RPE, interference score and serve accuracy are presented in Table 2. RPE post exercise tended to negatively correlate with serve accuracy (*r* = −0.57, *p* = 0.07; Figure 2A) and interference score of RT at post exercise (*r* = 0.65, p = 0.03; Figure 2B). The pre-to-post changes in second serve accuracy were negatively associated with the changes in interference score of RT (*r* = −0.54, *p* = 0.08; Figure 3A) and interference score of RT in the post exercise (*r* = −0.73, *p* = 0.01; Figure 3B).

**Table 2:** Relationship between RPE, interference score, and serve accuracy.

	Interference score RT at pre exercise	Interference score RT at post exercise	Change in interference score RT	Interference score correct reactions at pre exercise	Interference score correct reactions at post exercise	Change in interference score correct reactions	Serve accuracy at pre exercise	Serve accuracy at post exercise	Change in serve accuracy	HR at pre exercise	HR at post exercise	HRR	RPE
Interference score RT at pre exercise	–												
Interference score RT at post exercise	0.37	–											
Change in interference score RT	−0.52^+^	0.60^*^	–										
Interference score correct reactions at pre exercise	−0.42	−0.34	0.05	–									
Interference score correct reactions at post exercise	−0.36	−0.44	−0.10	0.33	–								
Change in interference score correct reactions	−0.09	−0.23	−0.13	−0.31	0.80^**^	–							
Serve accuracy at pre exercise	0.17	0.70^*^	0.50	−0.10	0.07	0.14	–						
Serve accuracy at post exercise	−0.08	−0.51	−0.40	0.39	0.42	0.18	−0.22	–					
Change in serve accuracy	−0.15	−0.73^*^	−0.54^+^	0.35	0.29	0.07	−0.65^*^	0.89^**^	–				
HR at pre exercise	−0.30	0.18	0.42	0.39	0.47	0.23	0.48	0.25	−0.03	–			
HR at post exercise	0.58^+^	0.64^*^	0.09	−0.38	−0.53^+^	−0.30	0.36	−0.47	−0.54^+^	0.12	–		
HRR	0.64^*^	0.60^+^	0.00	−0.46	−0.66^*^	−0.37	0.25	−0.56^+^	−0.56^+^	−0.16	0.96^**^	–	
RPE	0.48	0.65^*^	0.18	−0.60^*^	−0.90^**^	−0.52	0.04	−0.57^+^	−0.47	−0.41	0.67^*^	0.77^**^	–

** *p* < 0.01;

* *p* < 0.05;

+ *p* < 0.10.

## 4. Discussion

This study aimed to investigate the relationship between second serve accuracy and executive function in college tennis players. The results showed no significant change in RT in the congruent and incongruent conditions of the SCWT before and after the exercise. The rate of correct reactions did not change significantly in the congruent condition prior to and post the exercise, however they decreased significantly in the incongruent condition. Changes in second serve accuracy were negatively correlated with changes in interference score and interference score post the exercise.

### 4.1. Exercise intensity during exercise

Tennis players run approximately 8–15 m for one point and 3 m for each shot, on average, equating to a total of 1,300–3,600 m during a 1-h exercise session (Fernandez-Fernandez et al., 2009). Thus, tennis is a high-intensity activity. In this study, exercise intensity during exercise was evaluated using percentage of HRR, while the Borg scale was used to assess subjective exercise intensity. Additionally, rather than increasing the RPE using a match, physical exercise load was manipulated using tennis-specific exercise. The average subjective exercise intensity in previous studies using the Borg scale in actual tournaments and simulated games ranged from 11 (“light”) to 15 (“somewhat hard”), with a peak value of 17 (“very hard”; Mendez-Villanueva et al., 2007). The average value for subjective exercise intensity using the Borg scale was 14.4 post 60 min of exercise, with a peak value of 18 and a trough value of 12. Therefore, this study replicated the exercise intensity in a previous study that used tennis matches (Mendez-Villanueva et al., 2007).

### 4.2. Changes in result of SCWT pre and post exercise

RTs in the congruent and incongruent conditions did not change significantly between pre and post exercise conditions. However, the rate of the correct reactions index was significantly poorer. When completing this task, the participants were instructed to respond as quickly and accurately as possible. If participants had focused on providing all accurate responses, we may have observed no change in the number of wrong answers; however, their RTs may have been delayed. However, in this study, the participants may have focused on quick responses, and therefore, the number of wrong reactions increased as they could not make accurate judgments.

Brown and Bray (2015) reported that an increase in exercise intensity increased the number of false responses on the SCWT. Similarly, the results demonstrated the exercise was likely to have increased the number of false responses. Kamijo et al. (2004) reported an inverse U-shaped relationship between subjective exercise intensity and cognitive function. This previous study, using event-related brain potentials, reported that brain activity decreased during high-intensity exercise, and it may have occurred in the current exercise too. Additionally, the behavioral indices used in our study, such as RT of cognitive function and the number of wrong answers, only infer the information processing in the brain, and it is difficult to clearly separate whether the factor is related to the cognitive processing process or the response processing process. However, Hornery et al. (2007) reported that reproducibility of the serve action (e.g., position of the arm releasing the ball and of the ball) decreased with the passage of time. Similarly, the results demonstrated increased subjective exercise intensity may have affected the cognitive function, which in turn may have affected the response and processing process (e.g., serve movement).

### 4.3. Relationship between RPE, interference score, and serve accuracy

The change in the second serve accuracy before and after the exercise was negatively correlated with the interference score of RT and its change post-exercise. The results indicate that the change in the second serve accuracy decreased as the RT was delayed in the incongruent condition compared to the congruent condition. Therefore, the execution function may be one of the factors affecting the change in second serve accuracy.

In a previous study, athletes with higher subjective exercise intensity had lower second serve accuracy (Kuroda et al., 2016). Their second serve accuracy may have been influenced by physiological and psychological factors that the athlete experienced during the competition. Kuroda et al. (2016) examined the relationship between second serve accuracy and results of a grip strength test (muscle strength), spider run test (agility), and subjective evaluation (subjective feeling of fatigue and subjective exercise intensity) of university student tennis athletes. They reported that muscle strength and agility had no relationship with second serve accuracy, and they found a negative correlation with subjective exercise intensity. Additionally, Davey et al. (2002) found that the Loughborough test reduced stroke accuracy by 69% at exhaustion. Lyons et al. (2013) found that the Loughborough test (RPE = 18) reduced stroke accuracy by 40% at expert players and reported a 40% decrease in stroke accuracy with the Loughborough test (RPE = 18), and a decrease in athletic performance with increased subjective exercise intensity. Kamijo et al. (2004) reported an inverse U-shaped relationship between subjective exercise intensity and executive function. If the subjective exercise intensity is moderate, the executive function improves, but if subjective exercise intensity is low, the cognitive function may deteriorate. In addition, a recent study reported a relationship between executive function and sports performance (Ishihara et al., 2018). Ishihara et al. (2018) had university student tennis players play a simulated match and examined the relationship between statistics during the match and cognitive function. They showed the relationship between cognitive function and first serve points won as an index in a simulated match. Kuroda et al. (2016) experiment involved exercise in which subjective exercise intensity increased, which may have caused a decline in executive function and affected second serve accuracy. Therefore, it is possible that executive function may be a contributing factor to the poor performance in previous studies (Davey et al., 2002; Lyons et al., 2013).

The greatest strength of this study lies in its design. The present study is one of the few to examine the relationship between cognition and sport performance using a field-based approach. Most research in this area has been conducted in the laboratory. Therefore, this study extends previous findings on the relationship between exercise, cognition, and physical performance found in laboratory settings by suggesting practical applications to sport. Additionally, this study is one of the few to examine the relationship between changes in executive function over time and performance. Evidence indicating an association between executive function during games and sports performance is beneficial for athletes and their instructors.

Future research should examine whether executive function is truly related to athletic performance. Furthermore, we will examine whether improving the resting cognitive function improves tennis performance. It is also necessary to test whether improved resting cognitive function improves tennis performance and whether sustained or facilitative attention contributes to improved tennis performance. The present results provide the following practical implications. Second service accuracy is negatively associated with inhibitory function.

### 4.4. Limitation

There were only 11 participants. Given the sample size of 11 participants and a power = 0.80, the present study theoretically had sufficient sensitivity to detect an effect size exceeding *r* = 0.68 for the variance explained by each variable in the regression analysis. The main result of this study is the relationship between execution function and serve accuracy. Relevance of pre-to-post changes in second serve accuracy and interference score of RT in the post exercise were robust (*r* = −0.73, *p* = 0.01). However, relevance of the pre-to-post changes in second serve accuracy and the changes in interference score of RT were not robust (*r* = −0.54, *p* = 0.08). Thus, this result may not be applicable depending on the subject’s level of tennis competition. Therefore, it may not be possible to obtain robust conclusions from a small sample size. In the future, it is necessary to verify with rigorous, larger samples.

### 4.5. Conclusion

This study investigated the relationship between second serve accuracy and executive function in college tennis players. The results showed no significant change in RT in the congruent and incongruent conditions of the SCWT pre and post the exercise. The rate of correct reactions did not change significantly in the congruent condition pre and post the exercise, however, they decreased significantly in the incongruent condition. Changes in second serve accuracy were negatively correlated with interference score and its variations post the exercise. Future research should examine the relationship between the execution function and the actions proposed in previous studies that are related to the success of the serve (tossing up the ball) and selection of information (whether the toss position is appropriate for hitting the ball or not). Our results suggest that the time-course changes in executive function when playing tennis are negatively associated with the accuracy of the second serve. Thus, our findings expand previous knowledge regarding the positive association between time-course changes in executive functions and percentages in points won when playing tennis by including more specific skills (i.e., second serve accuracy).

## Data availability statement

The raw data supporting the conclusions of this article will be made available by the authors, without undue reservation.

## Ethics statement

The studies involving human participants were reviewed and approved by Hokusho University Research Ethics Committee. Written informed consent to participate in this study was provided by the participants’ legal guardian/next of kin.

## Author contributions

YK, TI, and MM participated in the design of the study and contribute to data collection and data analysis. YK contributed to writing manuscript, contributed to tennis competitive performance data collection, and contributed to the tennis competitive performance test design and interpretation of the results. TI contributed to executive function data collection. All authors contributed to the article and approved the submitted version.

## Funding

This study was supported by the Japan Society for the Promotion of Science (JSPS) KAKENHI Grant Numbers JP19K19981.

## Conflict of interest

The authors declare that the research was conducted in the absence of any commercial or financial relationships that could be construed as a potential conflict of interest.

## Publisher’s note

All claims expressed in this article are solely those of the authors and do not necessarily represent those of their affiliated organizations, or those of the publisher, the editors and the reviewers. Any product that may be evaluated in this article, or claim that may be made by its manufacturer, is not guaranteed or endorsed by the publisher.

## Abbreviations

HR: Heart rate
%HRR: Preliminary heart rate
RPE: Rating of perceived exertion
RT: Reaction time
SD: Standard deviation
SCWT: Stroop Color and Word Test
